# Complete mitochondrial genome of freshwater flea *Simocephalus sibiricus* Sars, 1899 (Crustacea: Cladocera: Anomopoda)

**DOI:** 10.1080/23802359.2021.1981787

**Published:** 2021-09-30

**Authors:** Yang-Liang Gu, Ping Liu, Bo-Ping Han

**Affiliations:** Department of Ecology, Jinan University, Guangzhou, China

**Keywords:** *Simocephalus sibiricus*, *Echinocaudus*, mitogenome, phylogenetic analysis

## Abstract

In this study, we presented the first complete mitochondrial genome of the genus *Simocephalus* determined by next-generation sequencing. The mitogenome of *S. sibiricus* is 15,818 bp in length, including 13 protein-coding genes (PCGs), two ribosomal RNAs, 22 tRNAs, and one putative control region, and has the same gene order with *Daphnia.* ATG and ATT were used as start codons in 11 PCGs, TTG was used in COX1 and GTG in ATP8. Six PCGs used an incomplete stop codon. Phylogenetic analysis based on 13 PCGs showed that, on genus level, *Simocephalus* was closely related to *Daphnia*.

The genus *Simocephalus* is a large group in Cladocera, commonly occurring in littoral zones of ponds and lakes. It was widely distributed but less studied (Forró et al. 2008; Xiang et al. 2015). The genus *Simocephalus* was reviewed by Orlova-Bienkowskaja (2001) and grouped into five subgenera: *Simocephalus* s. str., *Acutirostratus*, *Aquipiculus*, *Coroncephalus* and *Echinocaudus*. These subgenera were widely accepted by taxonomists, but many species in the genus *Simocephalus* were not (Kotov et al. 2013), e.g., *S. himalayensis* Chiang and Chen, 1974 and *S. himalayensis microdus* Chen, Shi and Shi, 1992. There were 14 species of *Simocephalus* recorded in China, but three of them were in the state of *incertae sedis* and two of them were considered as synonyms (Xiang et al. 2015). The first record of *S.* (*E.*) *sibiricus* Sars, 1898 in China was reported by Shi and Shi (1994) and distributed only in Heilongjiang Province (Shi and Shi 1996), which was established as a synonym of *S. exspinosus* later by Orlova-Bienkowskaja (2001). However, the validity of *S. sibiricus* was confirmed by genetic sequences (using *COXI* and *18S* sequences) and some morphological characters (short rhomboid ocellus, with 16–22 spines on the basal postabdomen claw, and the dorsal and ventral posterior valve margin with thick and strong denticles) (Huang et al. 2014). At present, there are only two mitogenomes of *Simocephalus* that were published on the NCBI database, i.e., one annotated partial mitogenome of *S. vetulus* (MT862434) and one non-annotated mitogenome of *S. serrulatus* (LS991523). In this study, we sequenced and annotated one complete mitogenome of *S. sibiricus* from southern China. It is also the first complete mitogenome for the genus *Simocephalus*, which will contribute to further study of the genus *Simocephalus* and the phylogeny of the Daphnidae.

The specimens of *Simocephalus* were collected in March 2021 from a pond in South China Botanical Garden (113.37°E, 23.19°N), Guangzhou City, southern China. The individuals of *Simocephalus* were identified as *S. sibiricus* with morphological characters of short rhomboid ocellus, around 20 spines on the basal postabdomen, thick and strong denticles on the dorsal ventral posterior valve margin according to Orlova-Bienkowskaja (2001) and Huang et al. (2014). The specimens of *S. sibiricus* were deposited at the Animal Specimens Museum of Jinan University (https://hydrobio.jnu.edu.cn/2021/0316/c29093a602403/page.htm, contact person: Ningning Liu; email: osss@jnu.edu.cn) under the voucher number ZOOPA07005. Around 150 specimens were picked out to extract the total DNA by using the TIANamp Marine Animals DNA Kit. The DNA was also stored at −80 °C at the Animal Specimens Museum of Jinan University. Genomic library was constructed by a high-throughput sequencing technology on Illumina's Novaseq family of platforms. We used the library and splicing software Novoplasty (https://github.com/ndierckx/NOVOPlasty) to conduct the *de novo* assembly of mitogenome with a COI sequence (KF960086) of *S. sibiricus* as seed. The annotation was based on the comparison annotation of NCBI mitochondrial database, with mitogenome of *S. vetulus* (GenBank Accession Number: MT862434) as seed.

The mitogenome of *S. sibiricus* (NO. MW848816) was 15,818 bp in length, containing 13 PCGs, two ribosomal RNA genes (rrnL and rrnS), 22 transfer RNA genes (tRNAs), and one putative control region. The gene order of *S. sibiricus* was consistent with that of daphnids. The nucleotide composition of the mitogenome has a significantly biased A + T content of 70.13% (A, G, C, and T was 33.93%, 14.30%, 15.57%, and 36.20%, respectively). The control region waslocated between tRNA-Ile and tRNA-Gln with a length of 1230 bp, and the A + T content was 73.17%. Four PCGs (*ND1*, *ND4*, *ND4L*, *ND5*), two rRNAs (*rrnL*, *rrnS*) and nine tRNAs (tRNA-Ile, tRNA-Gln, tRNA-Cys, tRNA-Tyr, tRNA-Phe, tRNA-His, tRNA-Pro, tRNA-Leu, and tRNA-Val) were transcribed from the L-strand, while the remaining 22 genes were encoded on the H-strand. ATG was used as the start codon in the most genes, i.e., *ATP6*, *COX2*, *COX3*, *CYTB*, *ND2*, *ND4,* and *ND5*. ATT was used as the start codon in *ND1*, *ND3*, *ND4L,* and *ND6*, TTG in *COX1,* and GTG in *ATP8*. Among 13 PCGs, seven ended up with typical stop codon of TAA or TAG, and six used an incomplete stop codon with single ‘T––’ (*COX1*, *COX2*, *ND3*, *ND4*, *ND5* and *CYTB*).

The phylogenetic tree was obtained using Bayesian Inference (BI) analysis based on entire protein coding genes of eleven Cladocera including the two *Simocephalus* species mentioned above (MT862434 and LS991523) and two outgroups (*Artemia tibetiana* and *A. salina*) in software BEAST v1.8.4, with GTR substitution model, MCMC chains of 100,000,000, and burn in 10%. The mitogenome of the *S. sibiricus* had similar A + T component with the other two *Simocephalus* species (69.2% for *S. vetulus* and 68.4% for *S. serrulatus*). The phylogenetic tree showed that the three *Simocephalus* species were in a cluster. On genus level, *Simocephalus* were closely related to *Daphnia* for the Daphniidae but was fully separated from Bosminidae (Figure 1). At a deeper level, the genus *Simocephalus* (order Anomopoda) was phylogenetically closer to the *Diaphanosoma* (order Ctenopoda) but greatly divergent from *Artemia* (order Anostraca). The phylogenetic relationships among the three orders (Anostraca, Ctenopoda, Anomopoda) and within Anomopoda in our study were identical to that of Schwentner et al. (2018) based on transcriptome data.

**Figure 1.**
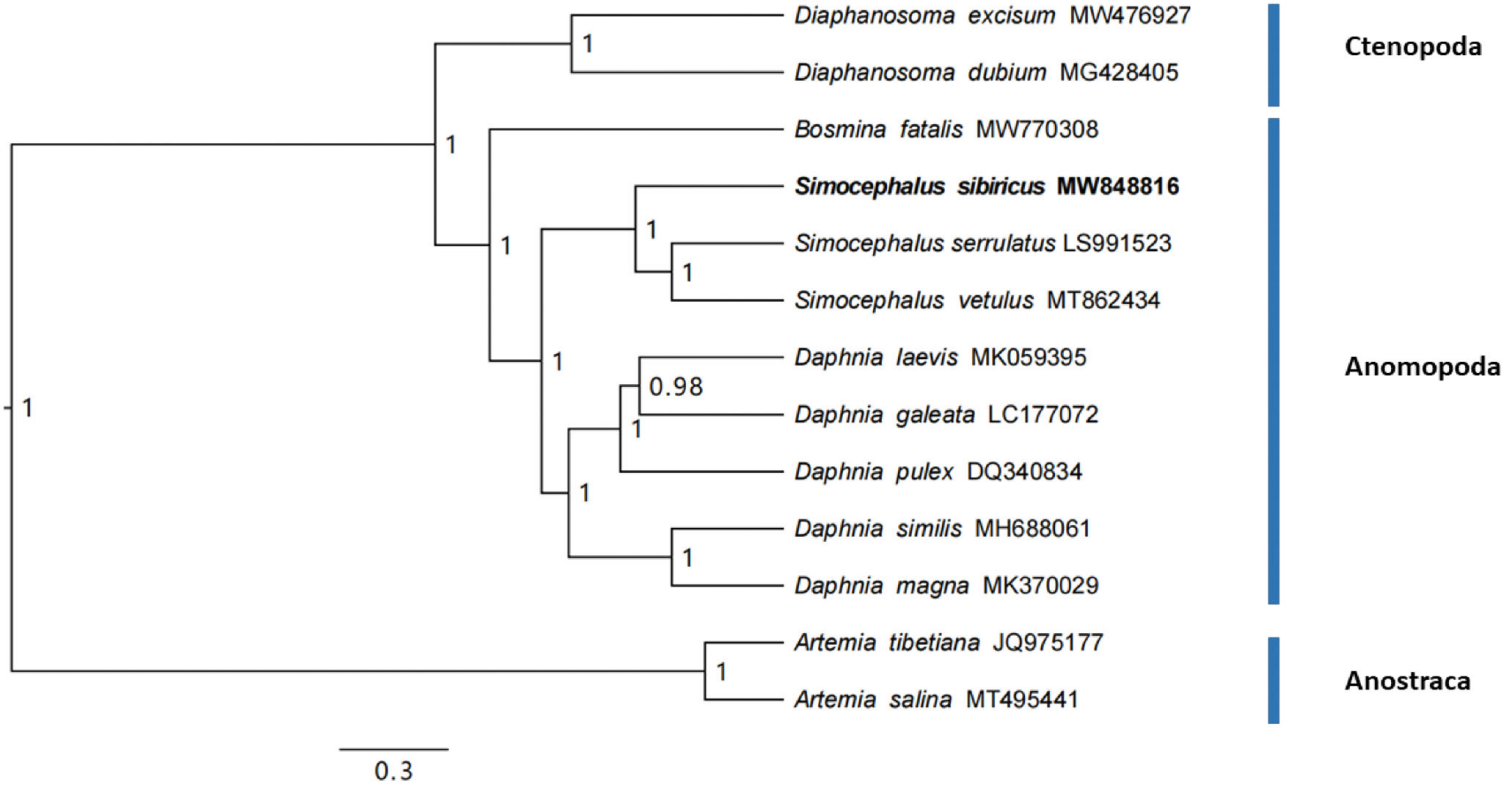
The phylogenetic tree was inferred with 13 PCGs of eleven Cladocera and two Anostraca that had partial/whole mitogenome in software BEAST v1.8.4, with GTR substitution model, MCMC chains of 100,000,000, and burn in 10%. Numbers on the nodes were posterior probability values.

## Data Availability

The genome sequence data that support the findings of this study are openly available in GenBank of NCBI at [https://www.ncbi.nlm.nih.gov] (https://www.ncbi.nlm.nih.gov) under the accession no. MW848816. The associated BioProject, SRA, and BioSample numbers are PRJNA719360, SRR1413763, and SAMN18603966 respectively.

## References

[CIT0003] Forró L, Korovchinsky NM, Kotov AA, Petrusek A. 2008. Global diversity of cladocerans (Cladocera; Crustacea) in freshwater. Hydrobiologia. 595(1):177–184.

[CIT0004] Huang XN, Shi XL, Kotov AA, Gu FK. 2014. Confirmation through genetic analysis of the existence of many local phyloclades of the genus *Simocephalus* (Crustacea, Cladocera) in China. PLoS One. 9(11):e112808. 25393020 10.1371/journal.pone.0112808 PMC4231159

[CIT0005] Kotov AA, Korovchinsky NM, Petrusek A. 2013. World checklist of freshwater Cladocera species. Available from: http://fada.biodiversity.be/group/show/17

[CIT0009] Orlova-Bienkowskaja MY. 2001. Cladocera, Anomopoda: Daphniidae, genus Simocephalus. Leiden: Backhuys; p. 130.

[CIT0010] Schwentner M, Richter S, Rogers DC, Giribet G. 2018. Tetraconatan phylogeny with special focus on Malacostraca and Branchiopoda: highlighting the strength of taxon-specific matrices in phylogenomics. Proc R Soc B. 285:20181524. 10.1098/rspb.2018.1524 PMC6125901 30135168

[CIT0011] Shi XL, Shi XB. 1994. On two new species and two new records of *Simocephalus* from China (Crustacea: Diplostraca: Daphniidae). Acta Zootaxonomica Sinica. 19:403–411.

[CIT0012] Shi XL, Shi XB. 1996. On the species and distribution of *Simocephalus* in Heilongjiang Province, China (Branchiopoda: Diplostraca). Acta Zootaxonomica Sinica. 21:263–276.

[CIT0013] Xiang XF, Ji GH, Chen SZ, Yu GL, Xu L, Han BP, Kotov AA, Dumont HJ. 2015. Annotated Checklist of Chinese Cladocera (Crustacea: Branchiopoda). Part I. Haplopoda, Ctenopoda, Onychopoda and Anomopoda (families Daphniidae, Moinidae, Bosminidae, Ilyocryptidae). Zootaxa. 3904(1):1–27. 25660769 10.11646/zootaxa.3904.1.1

